# Limits of normality of quantitative thoracic CT analysis

**DOI:** 10.1186/cc12738

**Published:** 2013-05-24

**Authors:** Massimo Cressoni, Elisabetta Gallazzi, Chiara Chiurazzi, Antonella Marino, Matteo Brioni, Federica Menga, Irene Cigada, Martina Amini, Alessandro Lemos, Marco Lazzerini, Eleonora Carlesso, Paolo Cadringher, Davide Chiumello, Luciano Gattinoni

**Affiliations:** 1 Dipartimento di Fisiopatologia Medico-Chirurgica e dei Trapianti, Università degli Studi di Milano, Via Francesco Sforza 35, 20122, Milano, Italy; 2 Dipartimento di Radiologia, Fondazione IRCCS Ca' Granda - Ospedale Maggiore Policlinico, Via Francesco Sforza 35, 20122, Milano, Italy; 3 Dipartimento di Anestesia, Rianimazione (Intensiva e Subintensiva) e Terapia del Dolore, Fondazione IRCCS Ca' Granda - Ospedale Maggiore Policlinico, Via Francesco Sforza 35, 20122, Milano, Italy

**Keywords:** acute lung injury, mechanical ventilation, computed tomography, CAT scan chest, lung

## Abstract

**Introduction:**

Although computed tomography (CT) is widely used to investigate different pathologies, quantitative data from normal populations are scarce. Reference values may be useful to estimate the anatomical or physiological changes induced by various diseases.

**Methods:**

We analyzed 100 helical CT scans taken for clinical purposes and referred as nonpathological by the radiologist. Profiles were manually outlined on each CT scan slice and each voxel was classified according to its gas/tissue ratio. For regional analysis, the lungs were divided into 10 sterno-vertebral levels.

**Results:**

We studied 53 males and 47 females (age 64 ± 13 years); males had a greater total lung volume, lung gas volume and lung tissue. Noninflated tissue averaged 7 ± 4% of the total lung weight, poorly inflated tissue averaged 18 ± 3%, normally inflated tissue averaged 65 ± 8% and overinflated tissue averaged 11 ± 7%. We found a significant correlation between lung weight and subject's height (*P *<0.0001, *r*^2 ^= 0.49); the total lung capacity in a supine position was 4,066 ± 1,190 ml, ~1,800 ml less than the predicted total lung capacity in a sitting position. Superimposed pressure averaged 2.6 ± 0.5 cmH_2_O.

**Conclusion:**

Subjects without lung disease present significant amounts of poorly inflated and overinflated tissue. Normal lung weight can be predicted from patient's height with reasonable confidence.

## Introduction

Computed tomography (CT) is the standard tool to understand the relationship between the anatomical basis and pathophysiology (gas exchange and mechanics) of acute lung injury/acute respiratory distress syndrome (ARDS) [1]. With computed tomography we measure the density of each voxel: assuming that lung is composed by two very different compartments, lung tissue with a density close to the water (0 Hounsfield Units, HU) and gas (-1000 HU), it is possible to measure gas volume and total lung weight as well as the degree of aeration associated with a given tissue fraction [2]. When helical CT is performed at different lung volumes/pressures it is also possible to explore the behavior of each tissue compartment using the lung pressure-volume curve [3]. This approach lead to pivotal advancements in the understanding of ARDS pathophysiology, such as the mechanisms of positive end-expiratory pressure (PEEP) [4] and prone positioning [5].

Unfortunately, reported quantitative helical CT scan data from normal populations - such as total lung weight, distribution of lung tissue or superimposed pressure - are scarce, and usually refer to few subjects [6]. The knowledge of reference values may be important to estimate, for example, lung edema and its distribution, which may be measured as excess tissue mass relative to normal subjects. The CT data from a normal population are therefore of potential interest. To answer this question we retrospectively collected from our database 100 subjects who underwent CT scan for clinical purposes.

We aimed at the extrapolation of total and regional lung tissue from anthropometric data (patient's height and sex) to obtain a predictive equation that can be applied to acute lung injury/ARDS patients.

## Materials and methods

### Study population

We retrospectively included in this study 100 subjects (53 males, 47 females), who underwent a helical lung CT scan for clinical purposes at Fondazione IRCCS Ca' Granda - Ospedale Maggiore Policlinico (Milan, Italy) and whose images were considered nonpathological by radiologists. CT scans were performed during a breath-hold at full inspiration (total lung capacity (TLC)) with the subject in a supine position.

The study was approved by the institutional review board of our hospital (Comitato di Etica della Fondazione IRCCS Ca' Granda Ospedale Maggiore Policlinico di Milano, no. 2959). Since the study was retrospective, the institutional review board of our hospital waived the necessity for collecting informed consent from patients.

Exclusion criteria were verified by patient history and by visual assessment of CT scan images: age <18 years; use of contrast enhancement for the examination's execution; situs inversus; previous pulmonary lobectomy/segmentectomy; lung nodules; and chronic or acute pulmonary diseases (pneumonia, interstitial pneumopathies, pulmonary fibrosis, pneumothorax, emphysema, chronic obstructive pulmonary disease, pulmonary tuberculosis, ARDS, lung cancer, pleural calcifications, pleural effusion, mesothelioma, pulmonary embolism).

Healthy subjects were those whose images were referred as nonpathological by radiologists.

The collection of morphometric data occurred in two phases: sex and age were recorded before CT execution, while height and weight were declared later by each subject, without the possibility to directly measure these parameters. Smoking status was available for 50% of patients.

### Computed tomography scanner

Images were acquired with a 128 detector Somatom Definition Flash (Siemens, Munich, Germany). The reconstruction gap was 5 mm (except for six subjects, in which the gap was 3 mm). The CT scan was set with a scan quality reference of 110 mA/second and 120 kV. Reconstruction algorithms were B70f (very sharp) for 54 subjects, B50f (sharp) for eight subjects, B31f for three subjects, and B40f for 23 subjects (smooth); the algorithm was not reported for 12 subjects.

### Image analysis

CT images were manually segmented by some of the authors (EG, CC, AM, MB, FM, IC) with dedicated software (Maluna 2.041 and Maluna 3.15, University of Mannheim, Germany), analyzed with other dedicated software (Soft-E-Film; http://www.elekton.it, Milan, Italy) and then revised by two radiologists (ML, AL).

### Quantitative analysis of the whole lung

We assumed that lung parenchyma is composed by two different compartments with very different densities: air with a CT number of -1000 HU and lung tissue with a CT number close to the water (0 HU) [1]. From the relation between CT number and density, it is possible to compute the tissue volume for each region of interest [1]:

CT number/-1,000=gas volume/(gas volume + tissue volume)

From the above equation, we can compute the following:

Tissue volume=(1-CT number/-1,000)×total volume

$$\text{Gas volume}=\left(\text{CT number}/-1,000\right)\times \text{total volume}$$

The total volume is the sum of the tissue volume and the gas volume.

Lung parenchyma was then classified into four compartments, according to the gas/tissue ratio [1]: noninflated tissue, density between +100 HU and -100 HU; poorly inflated tissue, density between -101 HU and -500 HU; well-inflated tissue, density between -501 HU and -900 HU; and overinflated tissue, density between -901 and -1,000 HU.

### Regional quantitative analysis

In each image along the apex-base axis, the lungs were divided into 10 sections. Each section was divided into 10 sterno-vertebral levels of equal height, as described by Pelosi and colleagues [6], obtaining 100 elements per lung, 200 elements per patient. The total height of each lung was measured as the distance from the ventral to the dorsal surface: level 1 refers to the most ventral region, while level 10 refers to the most dorsal. The height of each segment was measured as the distance from the most ventral to the most dorsal surface of the level in examination. Sterno-vertebral levels with the same number were merged (that is, all of the segments level 1 from apex to base, etc.), in order to obtain 10 regions for each lung (that is, 20 regions for each subject). The quantitative analysis performed for the whole lung was then executed for each region.

### Computation of superimposed pressure

We assumed that the lung behaves as a fluid-like structure where all the pressures are equally distributed. The superimposed pressure exerted on each lung element is equal to the density of the lung parenchyma above times the height of the parenchyma above:

$$\text{Superimposed pressure (cm}{\text{H}}_{\text{2}}\text{O})=\left(\text{lung tissue (g}\right)/\text{lung volume (ml}))\times \text{lung height (cm})$$

### Reference equations

Our data were compared with published predictive reference equations for functional residual capacity and TLC [7-12] (see Table 1).

**Table 1:** Predictive equations for total lung capacity and functional residual capacity

Equation	Females	Males
Cordero and colleagues [7]	TLC = 0.072 × h - 0.009 * A - 6.125	TLC = 0.106 × h - 1.396
Roberts and colleagues [10]	TLC = 7.107 × H - 6.435	TLC = 7.956 × H - 6.948
Roca and colleagues [11]	TLC = 92.687 × h + 8.301 * A - 9,129	TLC = 92.687 × h + 8.301 × A - 9,129
Quanjer and colleagues [9,12]	TLC = 6.60 × H - 5.79; FRC (sitting) = 2.24 × h + 0.01 × A - 1	TLC = 7.99 × H - 7.08; FRC (sitting) = 2.32 × h + 0.01 × A - 1.09
Ibanez and Raurich [8]	FRC (supine) = 1.39 × h - 0.424	FRC (supine) = 5.48 × h - 7.05

A, age (years); FRC, functional residual capacity; H, height (m); h, height (cm); TLC, total lung capacity.

### Statistical analysis

Data were analyzed with R Development Core Team (2010; Development Core Team (2011). R: A language and environment for statistical computing. R Foundation for Statistical Computing, Vienna, Austria. ISBN 3-900051-07-0, URL http://www.R-project.org/). All data are expressed as mean ± standard deviation, unless otherwise indicated. The relationship between patient's height, weight and age and CT scan parameters was assessed with linear regression, while males and females were compared using Student's *t *test. The Bland-Altman graphic method was used to evaluate the concordance between the CT scan gas volume and the predicted TLCs.

## Results

### Population characteristics

Table 2 summarizes the main demographic and CT scan characteristics of our population. As shown, males had a greater total lung volume, lung gas volume and lung tissue. We considered the total gas volume measured by CT scan, in spontaneously breathing subjects, at the end of a deep inspiration as their TLC, comprising both the residual volume and the vital capacity. When these values were normalized for height, these differences were confirmed (see Table S4 in Additional file 1). The frequency distributions of age, height and weight for our population are shown in Figures S9, S10 and S11 in Additional file 1.

**Table 2:** Demographic and computed tomography scan characteristics of the study population

	Population	Males	Females	*P *value
Patients (*n*)	100	53	47	
Patient weight (kg)	72 ± 14	78 ± 14	64 ± 11	<0.0001
Patient height (m)	1.7 ± 0.1	1.7 ± 0.1	1.6 ± 0.1	<0.0001
Body mass index (kg/m²)	25 ± 4	26 ± 4	24 ± 4	0.02
Age (years)	64 ± 13	63 ± 12	65 ± 14	0.64
Total lung volume (ml)	4,995 ± 1,313	5,757 ± 1,127	4,136 ± 923	<0.0001
Lung right	2,667 ± 691	3,063 ± 594	2,219 ± 492	<0.0001
Lung left	2,329 ± 659	2,694 ± 589	1,917 ± 466	<0.0001
Lung gas volume (ml)	4,066 ± 1,190	4,729 ± 1,033	3,319 ± 875	<0.0001
Lung right	2,174 ± 627	2,521 ± 545	1,784 ± 464	<0.0001
Lung left	1,892 ± 593	2,208 ± 534	1,535 ± 436	<0.0001
Lung weight (g)	929 ± 188	1,029 ± 168	817 ± 140	<0.0001
Lung right	492 ± 100	542 ± 89	435 ± 79	<0.0001
Lung left	435 ± 94	484 ± 86	380 ± 68	<0.0001
Over inflated tissue (%)	11 ± 7	12 ± 6	9 ± 7	0.03
Lung right	11 ± 7	12 ± 7	9 ± 8	0.05
Lung left	10 ± 7	12 ± 6	9 ± 7	0.04
Normal inflated tissue (%)	65 ± 8	64 ± 8	66 ± 8	0.12
Lung right	65 ± 8	64 ± 8	66 ± 9	0.14
Lung left	65 ± 8	64 ± 8	66 ± 9	0.10
Poorly inflated tissue (%)	18 ± 3	18 ± 3	18 ± 3	0.84
Lung right	18 ± 3	18 ± 3	18 ± 3	0.92
Lung left	18 ± 3	18 ± 3	18 ± 4	0.99
Not inflated tissue (%)	7 ± 4	6 ± 4	7 ± 4	0.53
Lung right	6 ± 4	6 ± 3	7 ± 4	0.56
Lung left	7 ± 4	7 ± 4	7 ± 4	0.74
Lung density (HU)	-805 ± 48	-816 ± 33	-792 ± 58	0.01
Lung right	-808 ± 46	-819 ± 32	-796 ± 56	0.01
Lung left	-804 ± 49	-815 ± 34	-791 ± 60	0.02
Average superimposed pressure (cm H_2_O)	2.6 ± 0.5	2.6 ± 0.6	2.6 ± 0.6	0.4
Lung right	2.6 ± 0.6	2.7 ± 0.5	2.6 ± 0.6	0.65
Lung left	2.7 ± 0.5	2.7 ± 0.4	2.6 ± 0.6	0.32
Transverse plane size (mm)	279 ± 27	298 ± 18	257 ± 17	<0.0001
Lung right	155 ± 29	165 ± 29	143 ± 25	<0.001
Lung left	136 ± 16	145 ± 12	127 ± 13	<0.0001
Coronal plane size (mm)	200 ± 22	214 ± 19	185 ± 13	<0.0001
Lung right	197 ± 22	210 ± 20	182 ± 13	<0.0001
Lung left	194 ± 21	206 ± 18	179 ± 13	<0.0001
Sagittal plane size (mm)	261 ± 26	270 ± 23	250 ± 25	<0.001
Lung right	254 ± 28	264 ± 24	243 ± 28	<0.001
Lung left	253 ± 28	262 ± 26	243 ± 28	<0.001

HU, Hounsfield units.

### Distribution of density

**Figure 1:**
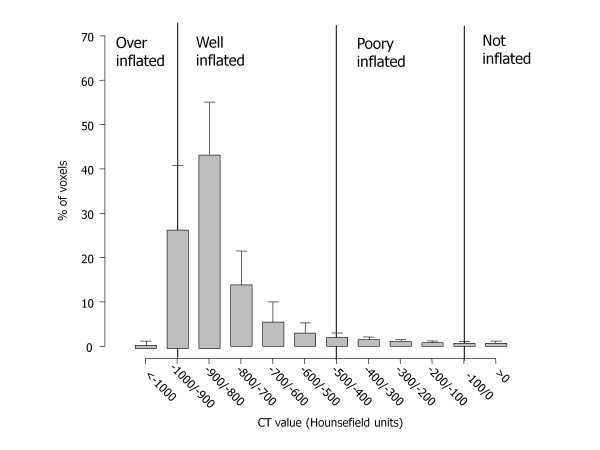
**Frequency distribution of Hounsfield unit numbers**. Frequency distribution of attenuation divided into intervals of 50 Hounsfield units (HU). Vertical lines indicate the ranges of lung inflation used in the literature: overinflated, between -1,000 and -901 HU; well aerated, between -900 and -501 HU; poorly aerated, between -500 and -101 HU; not aerated, <-100 HU. Note that frequency distribution refers to volume and not to weight. CT, computed tomography

Figure 1 shows the frequency distribution of lung parenchyma (HU). We measured an average 7 ± 4% of noninflated tissue, due to the inclusion of small bronchi and vessels, and a fraction of 18 ± 3% of poorly inflated tissue which, in part, may also be due to partial volume effects of the small bronchi, vessels, pleural interface or diaphragm; normally inflated tissue represented 65 ± 8% of the lung, while overinflated tissue included 11 ± 7% of lung parenchyma. One must note that these values refer to TLC.

#### Range of normality for lung weight

**Figure 2:**
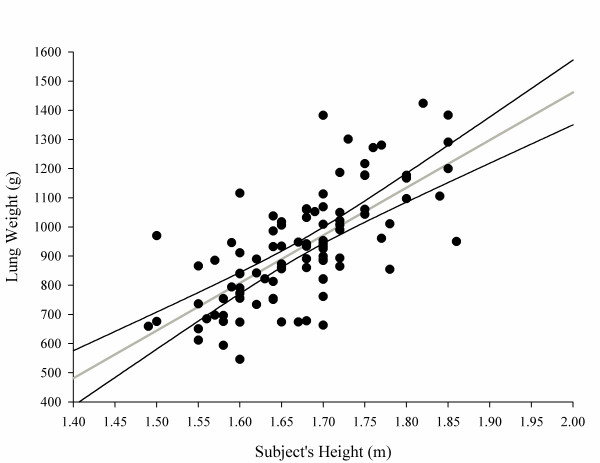
**Lung weight as a function of subject height**. Lung weight (g) = -1,806.1 + 1,633.7 × subject's height (m). The 2.5 to 97.5% confidence interval for intercept = -2,364.67 to -1,247.63; 2.5 to 97.5% confidence interval for slope = 1,300.53 to 1,966.93; *P *<0.0001, *r*^2 ^= 0.49.

Figure 2 shows the relationship between lung weight and subject's height in the whole population (*P *<0.0001, *r*^2 ^= 0.49):

Lung weight (g)=-1,806.1+1,633.7×subject's height (m)

**Figure 3:**
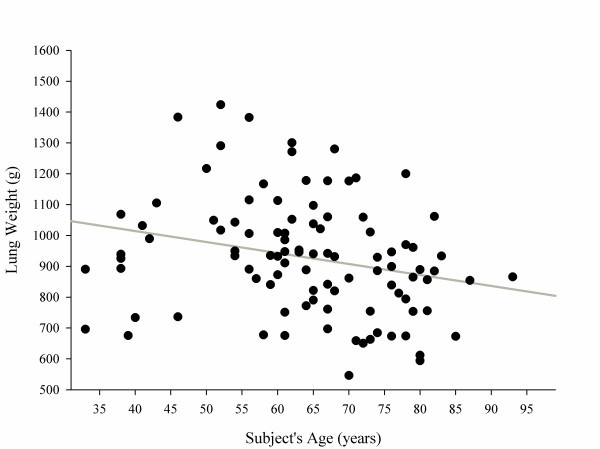
**Lung weight as a function of subject's age**. Lung weight (g) = 1,157.03 - 3.56 × subject's age (years). *P*= 0.01, *r*^2 ^= 0.06.

The regression slopes (*P *= 0.63) and intercepts (*P *= 0.56) of male sand females were not significantly different (see also Figure S12 in Additional file 1) for males and females separately. Surprisingly, we found a weak but negative relationship between lung weight and patient's age (Figure 3).

#### Determination of total lung capacity

**Figure 4:**
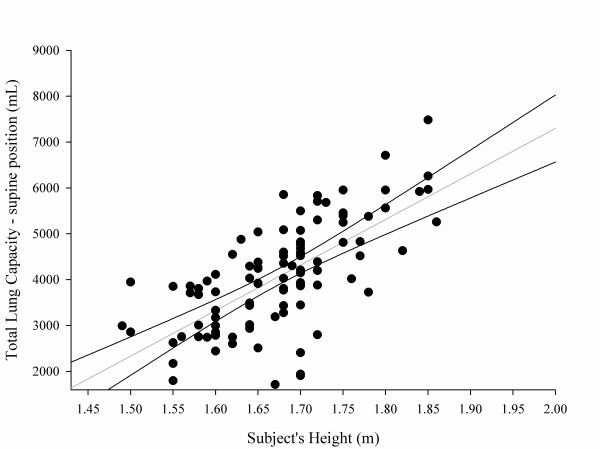
**Total lung capacity in a supine position as a function of patient height (m)**. Total lung capacity in a supine position (ml) = -12,550 + 9,924 × subject's height (m). The 2.5 to 97.5% confidence interval for intercept = 16,227 to 8,872; 2.5 to 97.5% confidence interval for slope = 7,730 to 12,118; *P *<0.0001, *r*^2 ^= 0.45.

**Figure 5:**
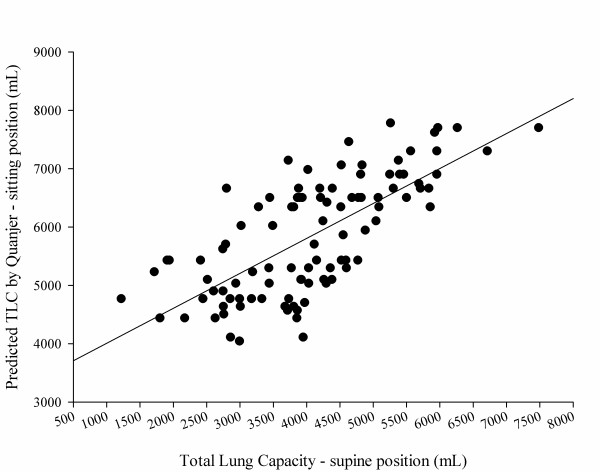
Total lung capacity in a sitting position predicted according to Quanjer and colleagues [9,12]. Total lung capacity (TLC) measured in a supine position as a function of computed tomography (CT) scan. **(A) **According to Quanjer and colleagues [9,12]: TLC (ml) (sitting) = 3,409 + 0.60 × CT scan (supine). *P *<0.0001, *r*^2 ^= 0.50. **(B) **Bland-Altman plot of the previous correlation. The average difference between the TLC predicted by Quanjer and colleagues [9,12] (sitting) and the CT scan TLC (supine) was 1,779 ± 849 ml (930 to 2,628). The difference between the predicted sitting TLC and the measured supine TLC decreased with the total gas volume increase. The difference between predicted TLC (Quanjer and colleagues) and measured TLC = 2,770 - 0.20 × (predicted TLC + measured TLC) / 2. *P *<0.001, *r*^2 ^= 0.05.

TLC in a supine position was related to subject's height (Figure 4) and weight but not subject's age (see Figures S13 and S14 in Additional file 1). Figure 5 presents the relationship, expressed both as linear regression and as a Bland-Altman plot, between the predicted TLC in a sitting position according to Quanjer and colleagues [9,12] and the measured TLC with CT scan in a supine position. Measured TLC in a supine position was ~1,800 ml lower than the predicted TLC in a sitting position; the difference between the predicted sitting TLC and the measured supine TLC decreased with the total gas volume increase. Similar results were obtained with different predictive equations (Table 3) (for the relationship between the various predictive equations, see Figures S15, S16 and S17 in Additional file 1).

**Table 3:** Relationship between measured and predicted gas volumes

Lung volume	Study	Estimated gas volume	Delta vs. measured supine gas volume	Estimated gas/tissue ratio	Estimated CT number (HU)
TLC, sitting position (ml)	Quanjer and colleagues [9,12]	5,501 ± 624	1,779 ± 849	6.4 ± 0.92	-863 ± 17
	Cordero and colleagues [7]	6,011 ± 1,025	1,899 ± 880	6.54 ± 1.02	-865 ± 18
	Roberts and colleagues [10]	6,008 ± 881	1,901 ± 845	6.56 ± 1.03	-865 ± 18
	Roca and colleagues [11]	6,960 ± 634	2,855 ± 886	7.67 ± 1.32	-882 ± 17
TLC, supine position (ml)	Measured	4,066 ± 1,190	-	4.42 ± 1	-805 ± 48
FRC, sitting position (ml)	Quanjer and colleagues [9,12]	3,160 ± 434	-950 ± 909	3.48 ± 0.54	-774 ± 27
FRC, supine position (ml)	Ibanez and Raurich [8]	2,126 ± 358	-1,984 ± 926	2.32 ± 0.35	-696 ± 31

CT, computed tomography; FRC, functional residual capacity; HU, Hounsfield units; TLC, total lung capacity.

### Regional distribution of gas and tissue

**Figure 6:**
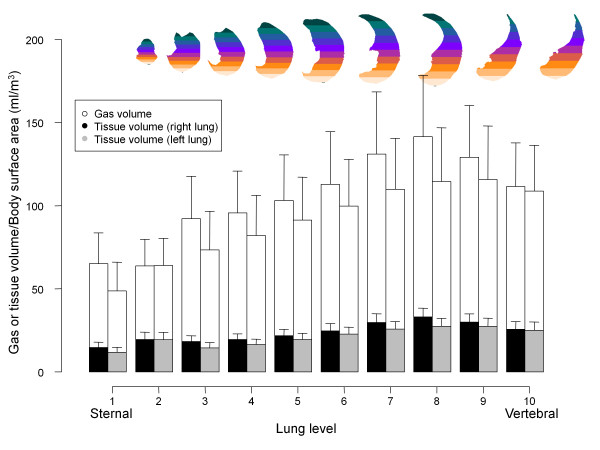
**Gas and tissue volume in 10 sterno-vertebral levels**. The sterno-vertebral distribution of gas (white) or tissue (gray for the right lung, black for the left lung) volumes, normalized for the body surface area (BSA). As the image shows, each lung was divided into 10 sterno-vertebral segments of equal height along the apex-base axis, as described by Pelosi and colleagues [6]. Sterno-vertebral levels with the same number were merged (that is, all of the segments level 1 from apex to base, etc.), in order to obtain 10 regions for each lung and then a quantitative analysis was performed.

**Figure 7:**
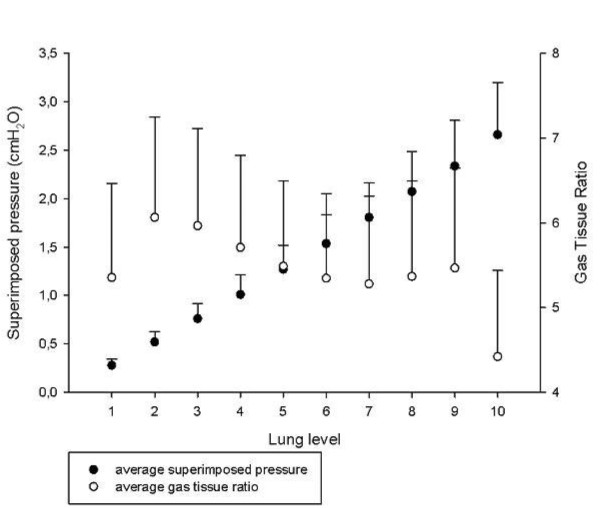
**Superimposed pressure and gas/tissue ratio as a function of the sterno-vertebral level**. Average superimposed pressure (filled circles) and the average gas/tissue ratio (empty circles) as a function of the sterno-vertebral level.

**Figure 8:**
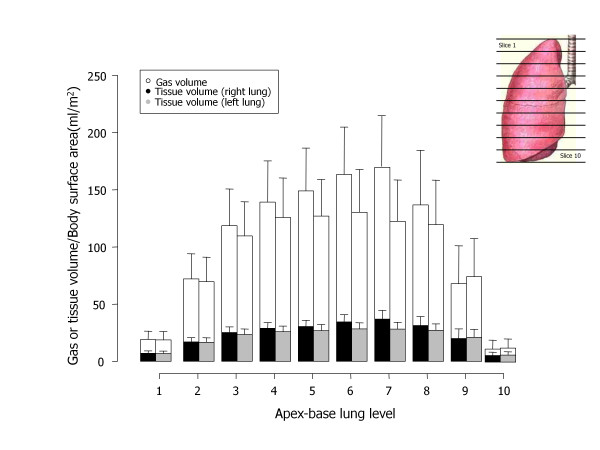
**Gas and tissue volume in 10 apex-base levels**. Apex-base distribution of gas (white) or tissue (gray for the right lung, black for the left lung) volumes, normalized for the body surface area (BSA); each lung was divided into 10 apex-base segments of equal height along the sterno-vertebral axis. Apex-base levels with the same number were merged (that is, all of the segments level 1, level 2, level 3, etc.), in order to obtain 10 regions for each lung and then a quantitative analysis was performed).

Figure 6 presents the distribution of gas and tissue volumes along the sternum-vertebral axis, which corresponds to an average superimposed pressure of 2.6 ± 0.5 cmH_2_O (see Figure 7 for the sternum-vertebral behavior of the superimposed pressure and the gas/tissue ratio). Figure 8 describes gas and tissue volumes going from apex to base.

## Discussion

We reported the limits of normality for quantitative CT scan analysis in a population of 100 subjects whose helical chest CT scan was reported as nonpathological by the attending radiologist. The data we report are different from the traditional densitometric data used for the quantitative assessment of emphysema or chronic obstructive pulmonary disease, since we focused on lung weight, its prediction and its distribution. We tried to extrapolate formulas to compute total lung weight, TLC in a supine position and regional tissue and gas volumes from patient's sex and height. Since the lung is an elastic inflatable object, the gas volume and the gas/tissue ratio we measured are dependent on the TLC status, while the lung weight and the noninflated tissue are not dependent on inflation, being the same at each point of the pressure-volume curve. The relationship between subject's height and lung weight was poorer than expected; in fact, prediction of total lung tissue only from subject's height and sex would not include other relevant anthropometric parameters such as body shape, which are extremely complex to quantitate. Other limitations of our CT study are concerned with biological factors, which include inspiration level, cardiac or diaphragmatic movements and co-morbidities [13].

The present study is different from traditional population databases that select only young and healthy subjects to define normal values, since no subjects were younger than 30 years and only 6% of subjects were younger than 50 years. We believe that our population is closer to hospitalized patients, who are usually old and with different co-morbidities (that is, many subjects underwent CT scan for cancer follow-up); mean age was 64 ± 13 years versus 55 ± 17 years for the patients who underwent CT scan for the determination of the potential for lung recruitment [14]. Data on smoking status were available only in 50 patients (50%), but we were unable to find any significant difference in CT scan values between smokers and nonsmokers (see Table 2 in Additional file 1). Patients with severe smoking-induced chronic obstructive pulmonary disease probably had a CT scan referred as pathological by attending radiologists and were excluded from our analysis. Of note, our population included very few subjects with pathological obesity and we therefore cannot exclude a selection bias since obesity is usually associated with other co-morbidities.

The distribution of lung parenchyma HU showed that only 65 ± 8% of lung parenchyma was classified as well inflated despite the CT scans being taken at TLC. A total 7 ± 4% of lung tissue was classified as having a gas/tissue ratio <0.1 or as noninflated. The noninflated fraction was similar to that obtained in 26 healthy piglets analyzed with the same methodology and imaged both at 0 end-expiratory pressure and 45 cmH_2_O inspiration (5 ± 2%) [15]. In total, 18 ± 3% of lung tissue was classified as poorly inflated despite imaging being performed at end inspiration: possible explanations may include partial volume effects or areas of fibrosis since the patient's average age was elevated. Even if patients were instructed to perform a full inspiration and hold their breath, there was no control on the maneuver: in fact, the percentage of poorly inflated tissue was related both to the total gas volume ( (*P *<0.0001, *r*^2 ^= 0.17):

Percentage of poorly inflated tissue=22-0.001×gas volume

and to the gas/tissue ratio (*P *<0.0001, *r*^2 ^= 0.31):

Percentage of poorly inflated tissue=25.10-1.63×gas tissue ratio

The fraction of overinflated tissue was 11 ± 7%, which appears quite high but one must remember that the original definition of the hyperinflated compartment was based on CT taken at functional residual capacity [1]; moreover, to explain this finding we may also take into account the presence of unrecognized areas of emphysema. One must stress that this result is relative to lung tissue and, since overinflated tissue has gas/tissue ratio >9, the overinflated volume accounts for a significant fraction of the total lung volume (27 ± 15%). Gevenois and colleagues performed a comparison between emphysema measured from the CT scans and from an anatomopathological analysis of lungs or lobes obtained from patients submitted for surgical resection for cancer or lung transplantation because of emphysema, suggesting that a CT scan threshold <-950 HU can be used to quantitate emphysema [16]. Using this threshold, we found a percentage of hyperinflated volume of 5.9 ± 5.4% in our subjects. Since this was a retrospective study, we did not perform respiratory function tests that would have provided information about the patient's functional status. Furthermore, it has been suggested that CT findings correlate with the presence and the severity of morphologic emphysema better than the results of pulmonary function tests [17].

Our study provides data regarding the average superimposed pressure in healthy lungs, which are compatible with the ones reported by Pelosi and colleagues on a single CT slice [6]. The increased superimposed pressure is the main mechanism of lung collapse in ARDS, and the PEEP works by counteracting the superimposed pressure [4]; in a healthy subject under anesthesia, therefore, a PEEP value ≤5 cmH_2_O should be sufficient to prevent the development dorsal atelectasies [18].

Different technical parameters of CT scan have been reported to influence the measurement of hyperinflated and nonaerated volumes [13,19]. The most important technical sources of noise are calibration, software for image analysis and the scanning protocol, which includes many elements, such as slice thickness, reconstruction algorithm and radiation dose [13]. Concerning the slice thickness, independent of the filter used, the hyperinflated volume increases markedly with decreasing thickness, while the nonaerated volume increases less, only 1% of the total volume, probably due to the reduced frequency of partial volume effects [20]. The slice thickness of our images was 5 mm except for six patients. Another possible source of variability is the reconstruction algorithm: different algorithms have been reported to determine statistically significant differences in lung volumes - a sharp algorithm is associated with an increase in hyperinflated lung volume [13,20,21]. However, when we tried to compare total lung volumes and hyperinflated volumes obtained with the very sharp algorithm (B70f) and the smooth one (B40f) in different patients, we did not find statistically significant differences. This study was conducted to estimate macroscopic data regarding the lung, such as the total lung weight, lung tissue distribution, along the apex-base or sternum-vertebral axis, and the total gas volume at end inspiration in a supine position; these parameters, regarding macroscopic regions of lung parenchyma, are unlikely to be affected by CT scan settings that operate mainly on the fine structure of the resulting images.

Some of our findings derive from the criteria used for the manual segmentation of lung parenchyma, which influence the results in two different ways: first, the arbitrariness of the criteria used (that is, criteria for bronchi/vessel exclusion, inclusion/exclusion of partial volume effects mainly at the apex and diaphragm or pleural surface, which depend also on the viewing window used for CT scan during manual segmentation); and second, on the inter-operator variability. The selection of a viewing window for manual lung segmentation is necessary since the number of gray tones distinguishable by the human eye is far lower than the ~2,000 levels of densities that the CT scan distinguishes between air and bone: an automated segmentation system may overcome this limitation with more uniform criteria for lung delimitation and bronchi/vessel exclusion.

In conclusion, our study suggests that in an unselected population the normal lung includes a physiologic 7% of noninflated tissue and relevant percentages of poorly inflated (18%) and hyperinflated tissue (11%), probably related to partial volume effects and to aging. We found a weak, but significant, relationship between total lung weight and age; we may speculate that this may be due to the loss of parenchyma and development of emphysema, which is part of the age-related reduction of lung function.

## Conclusion

The knowledge of reference values may be important to estimate, for example, the quantity of lung edema and its distribution, which may be measured as excess tissue mass, relative to normal subjects. The CT data from such populations are therefore of potential interest, in the perspective of better understanding ARDS pathophysiology and also the therapeutic mechanisms of PEEP and prone positioning.

The lung weight correlates well with the subject's height (*P *<0.0001, *r*^2 ^= 0.49):

Lung weight (g)=-1,806.1+1,633.7×subject's height (m)

while, surprisingly, there was a weak but negative relationship between lung weight and patient's age; furthermore, lungs of this aged population are characterized by a certain degree of inhomogeneity, with relevant amounts of poorly aerated tissue (18 ± 3%) and overaerated tissue (11 ± 7%). Considering the distribution of lung tissue and gas along 10 levels on the sternum-vertebral axis, it has been possible to calculate the superimposed pressure (2.6 ± 0.5 cmH_2_O).

## Key messages

• We chose a population that is closer to hospitalized patients, who are usually old and with different co-morbidities.

• Normal lung weight correlates well with subject's height.

• No relation was found between lung weight and subject's age.

• The normal lung in an aged population presents relevant amounts of poorly and overaerated tissue.

• The mean value of superimposed pressure in a normal lung is 2.6 ± 0.5 cmH_2_O.

## Abbreviations

ARDS, acute respiratory distress syndrome; CT, computed tomography; HU, Hounsfield units; PEEP, positive end-expiratory pressure; TLC, total lung capacity.

## Authors' contributions

LG is independent of any commercial funder, had full access to all of the data in the study, and takes responsibility for the integrity of the data and the accuracy of the data analysis. MC contributed to data analysis and drafted the manuscript. EG, CC and AM contributed to data acquisition, CT scan segmentation, analysis and writing the manuscript. MB, FM and IC contributed to data acquisition, CT scan segmentation and critically revising the manuscript.

MA critically revised the manuscript. AL and ML contributed to data acquisition, CT scan segmentation and critically revising the manuscript. EC contributed to data analysis and writing the manuscript. PC contributed to data acquisition, CT scan segmentation and critically revising the manuscript. DC contributed to the study concept, design and supervision, and critically revised the manuscript. LG contributed to the study concept, design and supervision, and writing the manuscript. All authors read and approved the final manuscript.

## Supplementary Material

Additional file 1 **Additional file 1: a Word document presenting additional methods and additional results**. Click here for file
