# Supplementary material for: Limits of normality of quantitative thoracic CT analysis
Source: Crit Care. 2013 May 24;17(3):R93. doi: 10.1186/cc12738 (PMC4057220; doi:10.1186/cc12738)
Supplement: Additional file 1 — a Word document presenting additional methods and additional results. [file cc12738-S1.DOC]

**Limits of normality of quantitative thoracic CT analysis**

- **Additional File -**

Massimo Cressoni^1^, Elisabetta Gallazzi^1^, Chiara Chiurazzi^1^, Antonella Marino^1^, Matteo Brioni^1^, Federica Menga^1^, Irene Cigada^1^, Martina Amini^1^, Alessandro Lemos^2^, Marco Lazzerini^2^, Eleonora Carlesso^1^, Paolo Cadringher^3^, Davide Chiumello^3^, Luciano Gattinoni^1,3^

^1^ Dipartimento di Fisiopatologia Medico-Chirurgica e dei Trapianti, Università degli Studi di Milano, Milan, Italy.

^2^Dipartimento di Radiologia, Fondazione IRCCS Ca’ Granda – Ospedale Maggiore Policlinico, Milan, Italy.

^3^Dipartimento di Anestesia, Rianimazione (Intensiva e Subintensiva) e Terapia del Dolore, Fondazione IRCCS Ca’ Granda – Ospedale Maggiore Policlinico, Milan, Italy.

**Additional methods**

Manual Segmentation

The manual segmentation of lung parenchima was performed in the sequent way: we followed the outline of each lung displayed in the mediastinal window (CT min = -250 HU, CT max = +150 HU) following the inner border of the rib and the external border of the mediastinum, while we preferred the Full CT – scale window (CT min = -1000 HU, CT max = +1000 HU) to view bronchi, bronchioles and blood vessels, which allowed to identify these structures in a more accurate and easy way, employing their different densities. We chose to exclude the main bronchi (or primary bronchi), lobar bronchi (or secondary bronchi), segmental bronchi (or tertiary bronchi), while bronchioles were usually included in the regions of interest due to their small dimensions (which made their identification and manual segmentation arduous). The diaphragm was excluded using the mediastinal window.

Regional quantitative analysis

Each image along the apex-base axis was assigned to the right or the left lung, computing the barycenter of the polygon which inscribes the lung as follows:

- Barycentrum x axis: summation of x axis coordinates/number of voxel
- Barycentrum y axis: summation of y axis coordinates/number of voxel

Determination of superimposed pressure

Assuming that pressure is transmitted to the parenchimal dependent regions as a fluid, it is possible to calculate, in each sterno-vertebral level, the pressure determined by the overlying tissue compared to the level in examination. This pressure, defined superimposed pressure, is obtained multiplying the density for the height of each level.

Hydrostatic pressure (P) of each segment is computed as follows:

P = segment density (g/ml) * segment height (cm)

segment density (g/ml) = tissue in segment (g)/segment volume (ml)

the amount of tissue in each voxel is computed as:

tissue (g) = (1 – CT/-1000)*voxel volume (ml)

and the amount of tissue in the segment is the sum of all tissue volumes included in the segment while the segment volume is the sum of the volumes of all voxels included into the segment.

Superimposed pressure (Sp) of one level is computed adding hydrostatic pressure (P) of that level to the hydrostatic pressures of the levels above. For instance, superimposed pressure (Sp) at level 7 is the sum of all the hydrostatic pressures P from level 1 to 7. Total superimposed pressure (SP_T_) is the superimposed pressure in the most dorsal level, i.e. level 10.

**Additional Results**

**Table S4: Characteristics of the studied population normalized for the patients’ height.**

|  | **Population** | **Male** | **Female** | **P** |
| --- | --- | --- | --- | --- |
| **Total Lung Volume Normalized (mL)** | 2964±680 | 2549±539 | 3333±572 | < 0.0001 |
| **Lung Gas Volume Normalized (mL)** | 2412±629 | 2045±518 | 2736±535 | < 0.0001 |
| **Lung Weight Normalized (g)** | 553±94 | 503±79 | 596±86 | < 0.0001 |

**Table S5:** Characteristics of the studied population distinguished in smokers and not-smokers.

|  | **Population** | **Smokers** | **Not smokers** | **P** |
| --- | --- | --- | --- | --- |
| **N. subjects** | 50 | 17 (34%) | 33 (66%) |  |
| **Patients' weight (kg)** | 72 ± 15 | 71 ± 16 | 73 ± 15 | 0.76 |
| **Patients' height (m)** | 1.68 ± 0.07 | 1.67 ± 0.06 | 1.68 ± 0.07 | 0.57 |
| **BMI (kg/m²)** | 25 ± 4 | 25 ± 4 | 25 ± 4 | 0.88 |
| **Age (years)** | 62 ± 14 | 54 ± 11 | 66 ± 13 | <0.005 |
| **Total Lung Volume (mL)** | 5137 ± 1159 | 4969 ± 1269 | 5224 ± 1109 | 0.49 |
| **Lung Gas Volume (mL)** | 4198 ± 1032 | 4018 ± 1134 | 4290 ± 981 | 0.41 |
| **Lung Weight (g)** | 940 ± 195 | 950 ± 226 | 934 ± 181 | 0.8 |
| **Over inflated tissue (%)** | 11 ± 6 | 9 ± 6 | 12 ± 6 | 0.05 |
| **Normal inflated tissue (%)** | 66 ± 7 | 70 ± 7 | 64 ± 6 | 0.003 |
| **Poorly inflated tissue (%)** | 17 ± 3 | 16 ± 4 | 18 ± 2 | 0.14 |
| **Not inflated tissue (%)** | 6 ± 3 | 5 ± 3 | 6 ± 3 | 0.14 |
| **Lung density (HU)** | -811 ± 38 | -801 ± 52 | -816 ± 27 | 0.28 |
| **Average superimposed pressure (cmH₂O)** | 2.6 ± 0.5 | 2.6 ± 0.6 | 2.5 ± 0.4 | 0.58 |
| **Trasverse plane size (mm)** | 280 ± 26 | 276 ± 25 | 283 ± 26 | 0.42 |
| **Coronal plane size (mm)** | 198 ± 23 | 194 ± 27 | 200 ± 21 | 0.45 |
| **Sagittal plane size (mm)** | 264 ± 23 | 259 ± 19 | 267 ± 24 | 0.25 |

**Figure S9.**

**Figure S10**.

**Figure S11.**

**Figure S12.**


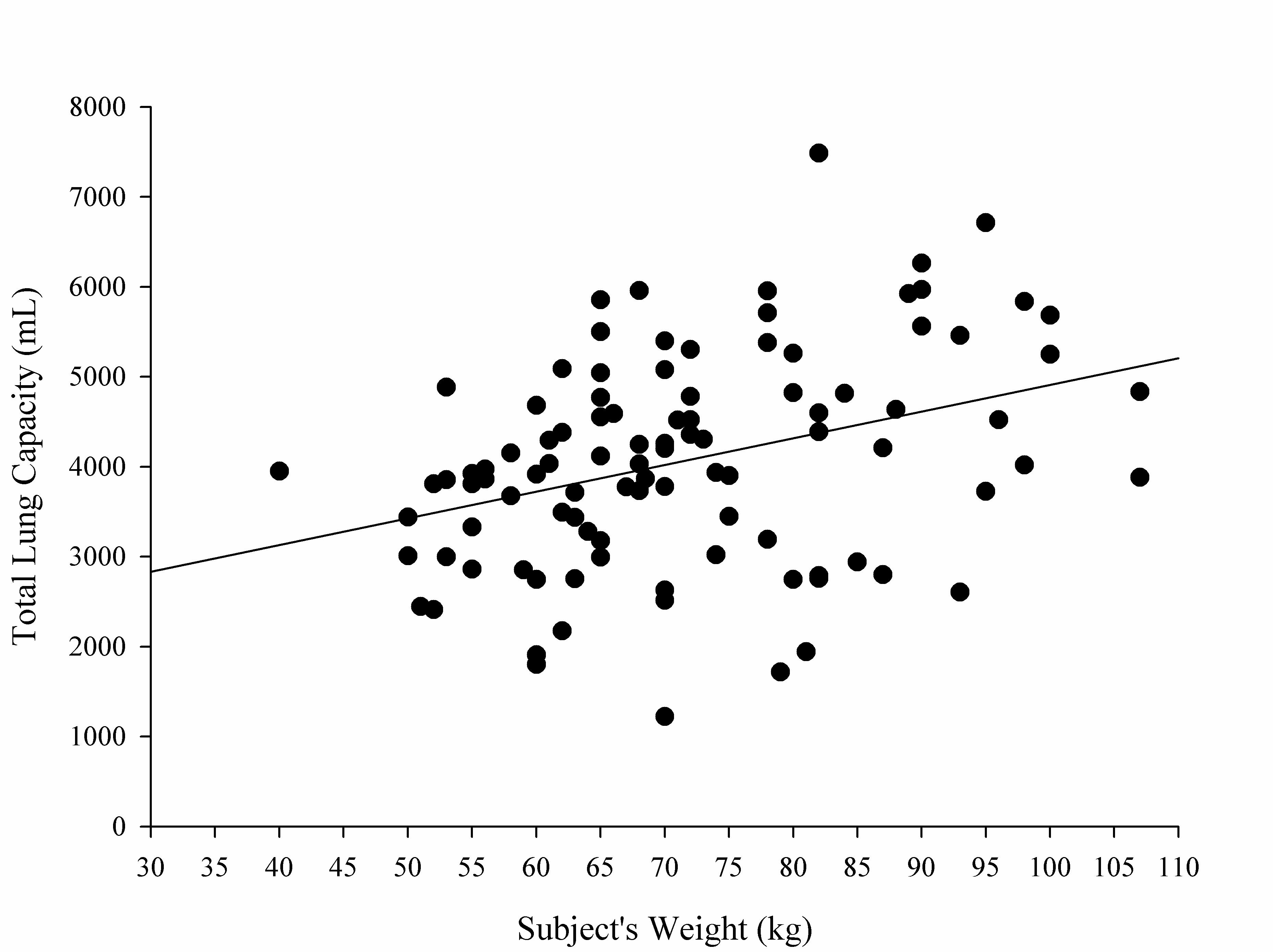


**Figure S13.**


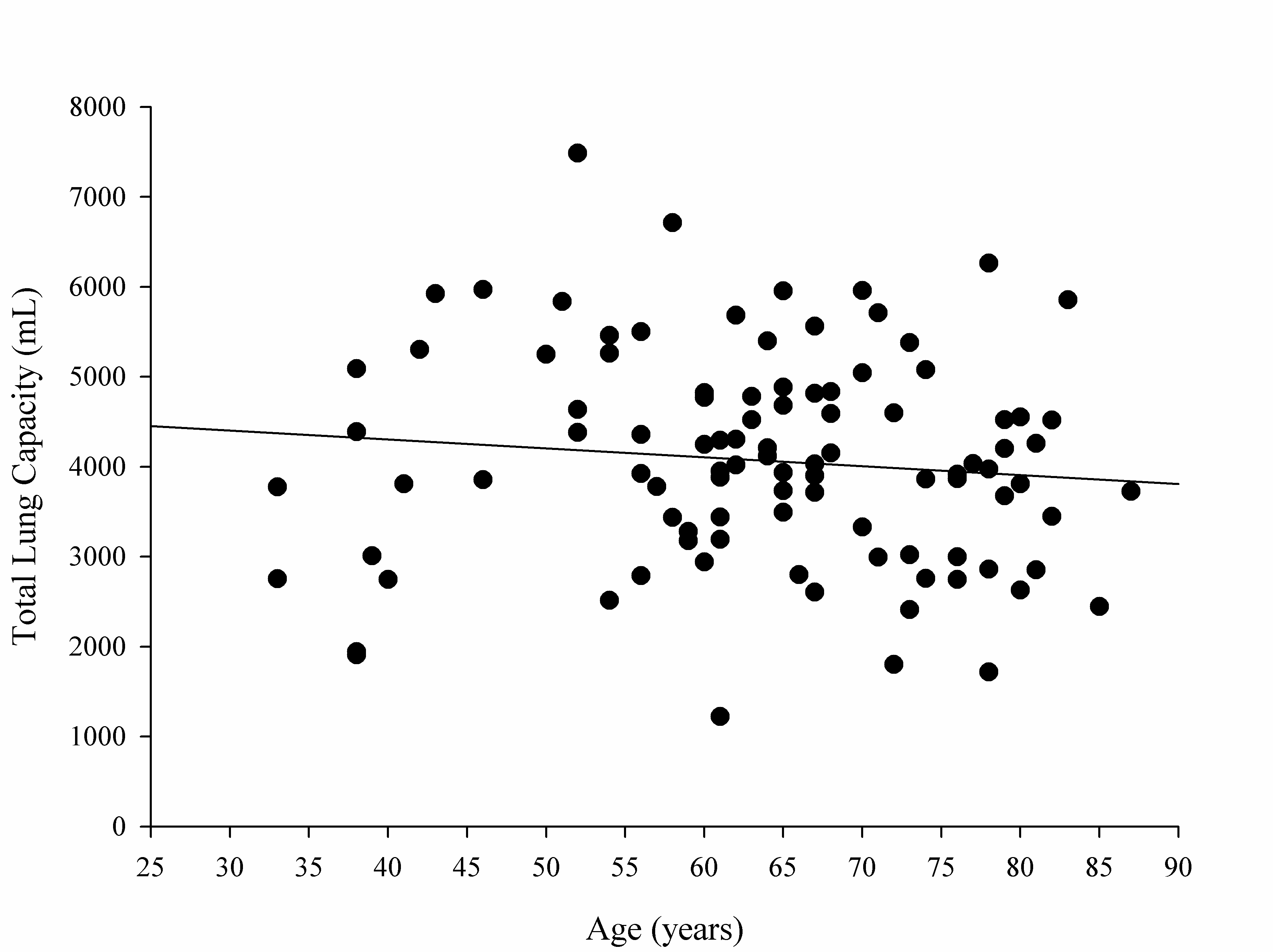


**Figure S14.**


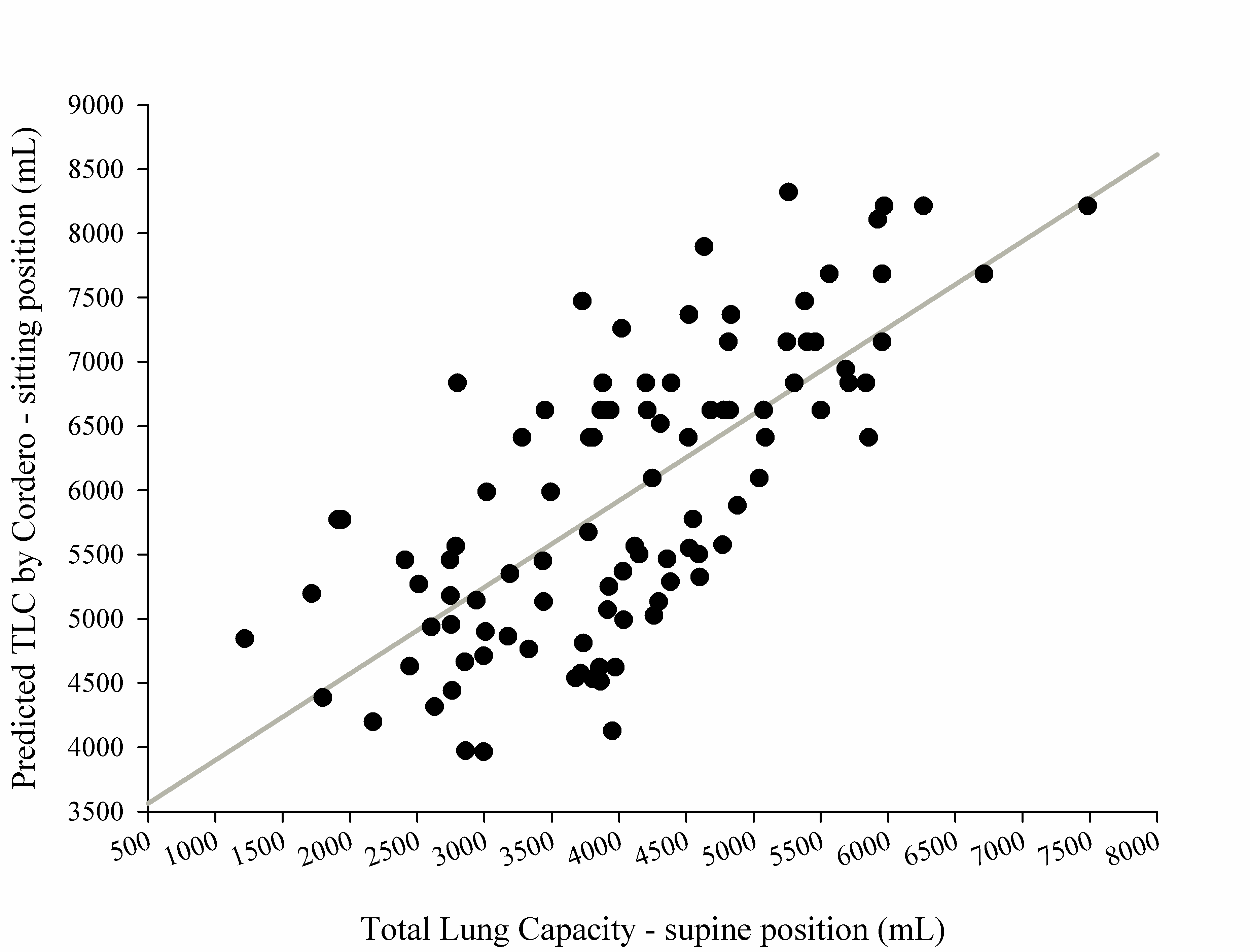


**Figure S15 Panel A.**


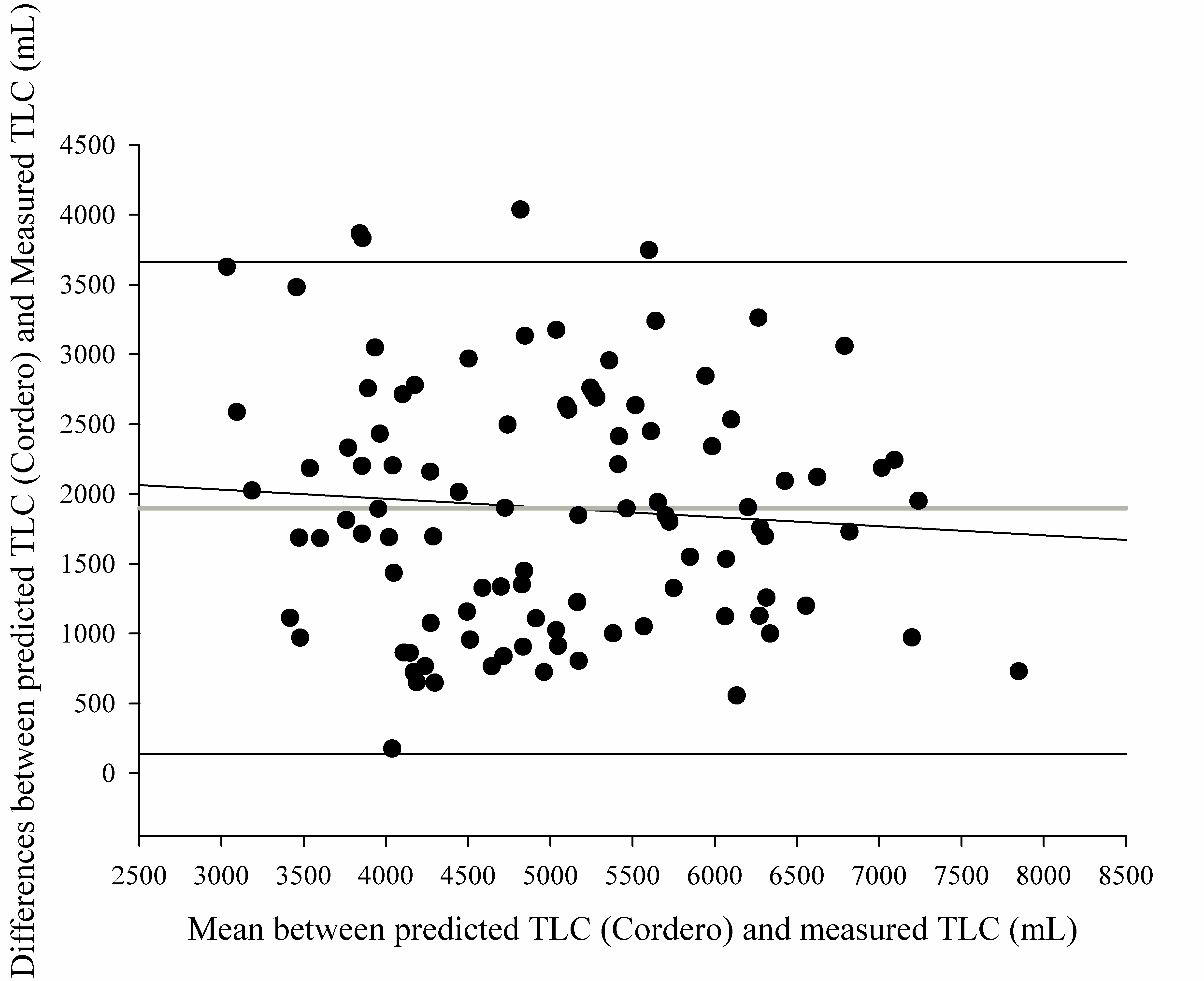


**Figure S15 Panel B.**


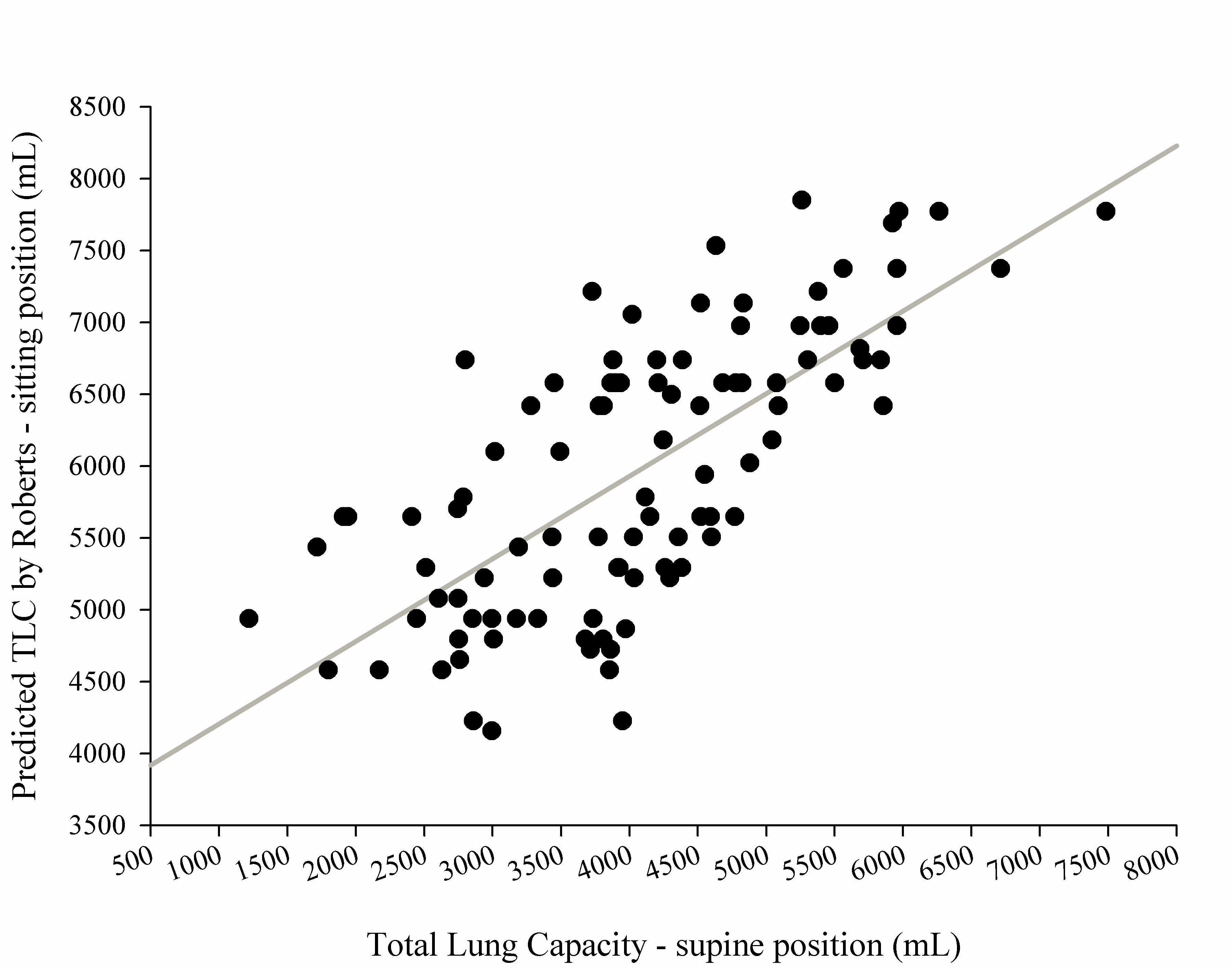


**Figure S16 Panel A.**


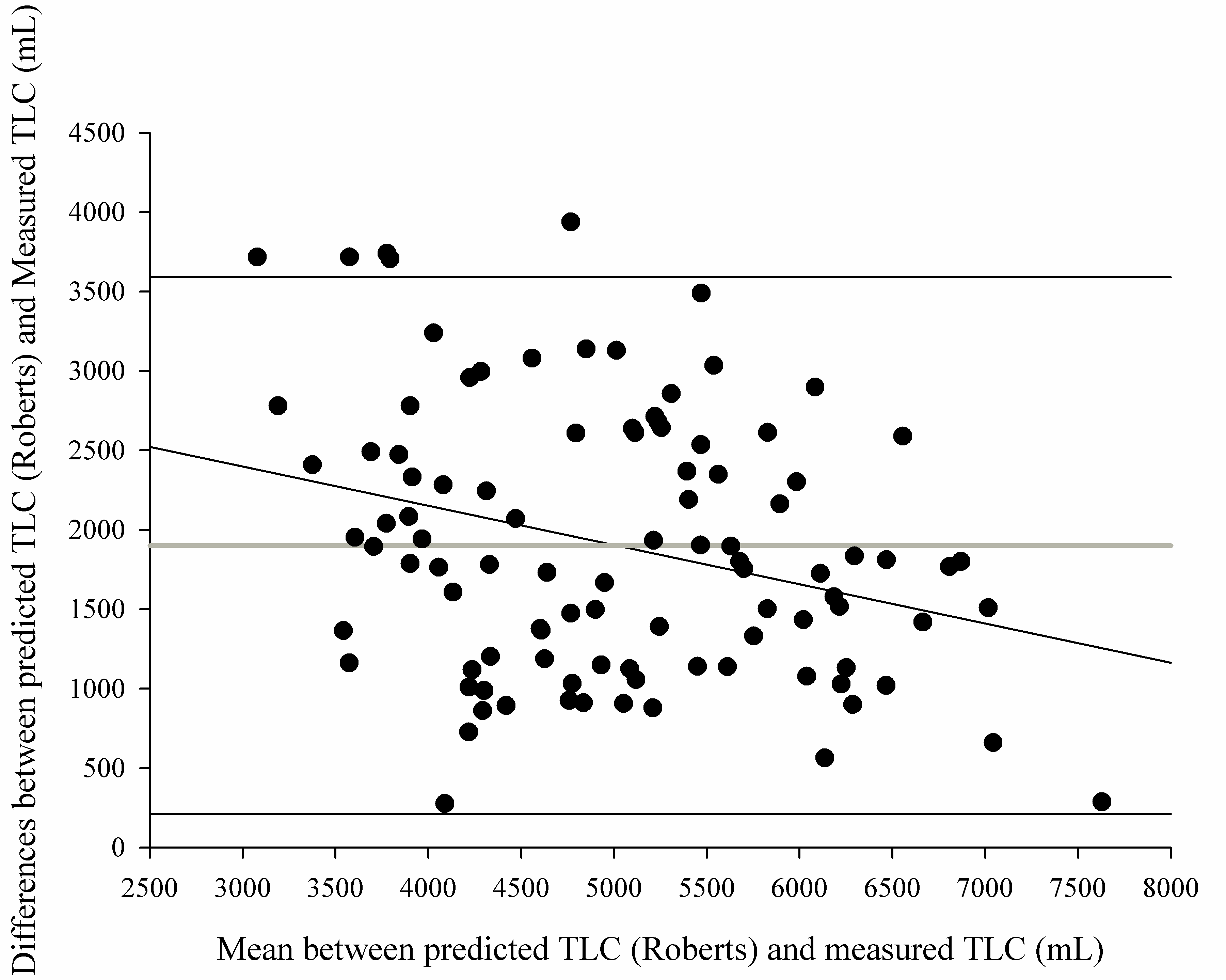


**Figure S16 Panel B.**


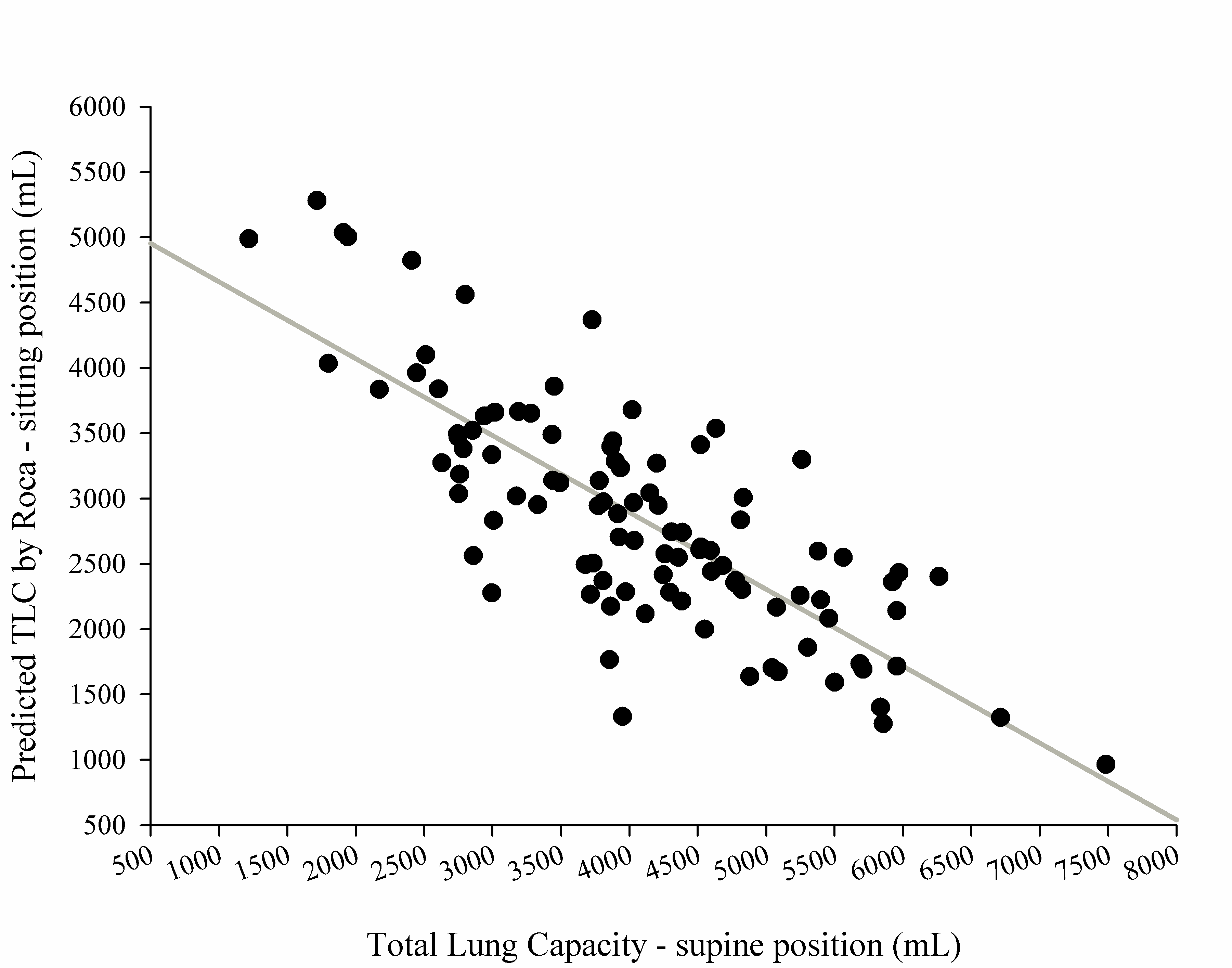


**Figure S17 Panel A.**


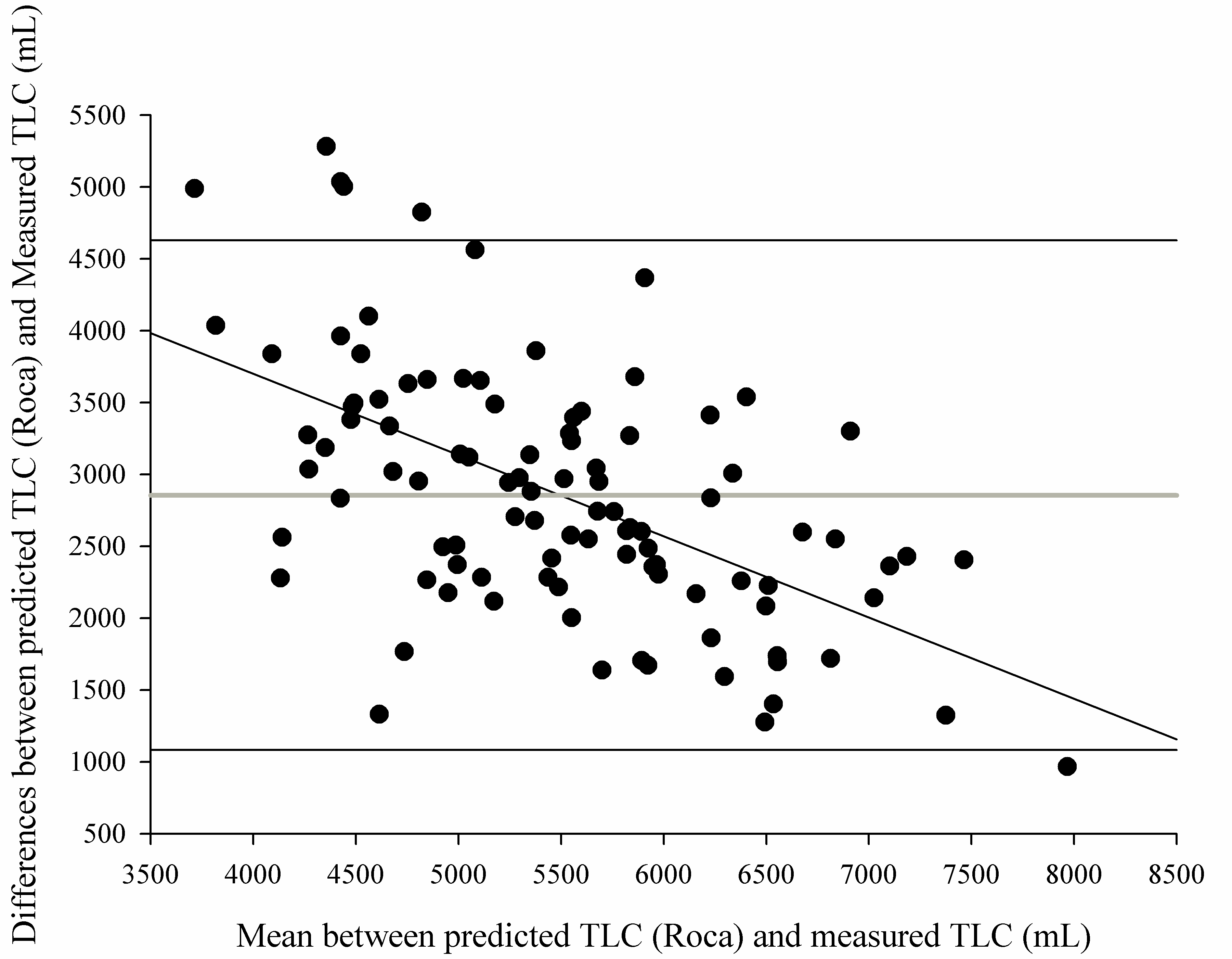


**Figure S17 Panel B.**



**Figure legends**

**Figure S9:** Frequency distribution of subjects’ age (years).

The figure describes the frequency distribution of subjects’ age divided in intervals of 10 years.

**Figure S10:** Frequency distribution of subjects’ height (m).

The figure describes the frequency distribution of subjects’ height divided in intervals of 10 cm.

**Figure S11:** Frequency distribution of subjects’ weight (kg).

The figure describes the frequency distribution of subjects’ weight divided in intervals of 10 kg.

**Figure S12**: Lung weight (g) as a function of subjects' height (m).

Right panel – males: lung weight = -1438 + 1433*height (m) [2.5% - 97.5% confidence interval for

intercept -2436 – -440; 2.5% - 97.5% confidence interval for slope 853 - 2012] , r2=0.31, p<0.0001

Left panel – females: lung weight = -1093 + 1178*height (m) [2.5% - 97.5% confidence interval for

intercept -2108 – -77; 2.5% - 97.5% confidence interval for slope 552 - 1084] , r2=0.23, p<0.001 97x40mm (300 x 300 DPI)

**Figure S13:** Total lung capacity (ml) as function of subject's weight (Kg).

TLC (ml) = 1941 + 29.67*Weight (Kg), r^2^=0.12, p <0.001

**Figure S14:** Subject's age (years) as function of Total Lung Capacity measured in supine position (mL).

Total lung capacity (ml) = 4699 -9.884 * Subject's age, p=0.28, r² = 0.00

**Figure S15:** Total Lung Capacity predicted in sitting position (mL) according to Cordero [1]as function of Total Lung Capacity measured in supine position (mL).

**Panel A:** Total Lung Capacity predicted in sitting position (mL) according to Cordero[1] = 3227 * Total Lung Capacity * 0.67 measured in supine position (mL), p<0.0001, r² = 0.50

**Panel B:** Bland e Altman plot of the previously described correlation.

The average difference between the TLC predicted by Cordero[1] (sitting) and the CT scan TLC (supine) was 1899 ml [1019 – 2779]. The average difference did not depend on the absolute lung volume.

**Figure S16:** Total Lung Capacity predicted in sitting position (mL) according to Roberts [2]as function of Total Lung Capacity measured in supine position (mL).

**Panel A:** Total Lung Capacity predicted in sitting position (mL) according to Roberts[2] = 3631 + 0.57 * Total Lung Capacity measured in supine position (mL), p<0.0001, r² = 0.50

**Panel B:** The figure shows the Bland e Altman plot of the previously described correlation.

The average difference between the TLC predicted by Roberts[2] (sitting) and the CT scan TLC (supine) was 1901 ml [1057 – 2745]. The bias between the two methods was significantly related to the absolute lung volume (Difference between the TLC predicted by Roberts[2] (sitting) and the CT scan TLC (supine) = 3141 – 0.25*average between between the TLC predicted by Roberts[2] (sitting) and the CT scan TLC (supine), r^2^ = 0.08, p <0.001).

**Figure S17:** Total Lung Capacity predicted in sitting position (mL) according to Roca[3] as function of Total Lung Capacity measured in supine position (mL).

**Panel A:** Total Lung Capacity predicted in sitting position (mL) according to Roca[3]= 5248 + 0.41 * Total Lung Capacity measured in supine position (mL), p<0.0001, r² = 0.44

**Panel B:** The figure shows the Bland e Altman plot of the previously described correlation.

The average difference between the TLC predicted by Roca (sitting) and the CT scan TLC (supine) was 2855 ml [1969-3741]. The bias between the two methods was significantly related to the absolute lung volume (Difference between the TLC predicted by Roca[3] (sitting) and the CT scan TLC (supine) = 5961 – 0.57*average between between the TLC predicted by Roca[3] (sitting) and the CT scan TLC (supine), r^2^ = 0.31, p <0.

References

1. Cordero PJ, Morales P, Benlloch E. **Static lung volumes: reference values from a Latin population of Spanish descent**. Respiration 1999 66:242-250

2. Roberts CM, MacRae KD, Winning AJ **Reference values and prediction equations for normal lung function in a non-smoking white urban population**. Thorax, 1991 46:643-650

3. Roca J, Burgos F, Barbera JA. **Prediction equations for plethysmographic lung volumes**. Respir Med, 1998 92:454-460
